# Academic expectations stress, its associations with symptoms of depression and anxiety, and preliminary exploration of suicidality: results from a National Youth Mental Health Survey in Singapore

**DOI:** 10.3389/fpsyg.2026.1785980

**Published:** 2026-05-28

**Authors:** Yi Chian Chua, Charmaine Tang, Bernard Tan, Yi Ping Lee, Janhavi Ajit Vaingankar, Sherilyn Chang, Yeow Wee Brian Tan, Ellaisha Samari, S. Archana, Rebecca P. Ang, Mythily Subramaniam, Swapna Kamal Verma

**Affiliations:** 1 Department of Psychosis, Institute of Mental Health, Singapore, Singapore; 2 Research Division, Institute of Mental Health, Singapore, Singapore; 3 CHAT, Center of Excellence in Youth Mental Health, Institute of Mental Health, Singapore, Singapore; 4 Psychology and Child & Human Development Department, National Institute of Education, Nanyang Technological University, Singapore, Singapore; 5 Saw Swee Hock School of Public Health, National University of Singapore, Singapore, Singapore; 6 Lee Kong Chian School of Medicine, Nanyang Technological University, Singapore, Singapore; 7 Medical Board, Institute of Mental Health, Singapore, Singapore; 8 MD Programme Department, Duke-NUS Medical School, Singapore, Singapore

**Keywords:** academic expectations stress, anxiety, depression, suicidality, youth

## Abstract

**Background:**

The Academic Expectations Stress Inventory (AESI) was developed to measure levels of academic stress resulting from perceived expectations from parents and teachers, and expectations from the self, among middle and high school students. The objectives of the present study are to describe AESI scores and compare its total and subscale scores among youths in Singapore, investigate relevant sociodemographic correlates, and examine the association between AESI scores and symptoms of depression and anxiety, with a brief exploration on suicidality.

**Methods:**

Data analyzed for this study was collected as part of a larger study—the National Youth Mental Health Study, a cross-sectional nationwide survey of youths aged 15–35 years. Participants were recruited through door knocks and street intercepts. The survey contained a battery of self-administered instruments, including a sociodemographic section, the Depression Anxiety Stress Scales 21, and the AESI. Nearing the tail-end of the study, a brief questionnaire on suicidality was added and administered to a subset of participants.

**Results:**

Of the total 2600 participants who were recruited for the original study, 959 respondents indicated that they were “currently studying”. On the AESI total scale, age group, gender, ethnicity, monthly household income, paternal education, and number of siblings remained significant correlates in the regression, after accounting for other sociodemographic variables. AESI scores were significantly associated with severe depression and anxiety. A logistic regression revealed that the AESI total score remained significantly associated with having at least one suicidal ideation or behavior in the past 12 months.

**Conclusions:**

In sum, the data seems to suggest that being female, being an only child, and belonging to the middle-income group are associated with increased risk of greater academic stress, particularly due to expectations from the self. It is hoped that results from this study would inform future interventions targeted at managing academic expectations and preventing suicide among youth.

## 1 Introduction

Stress associated with academic expectations has been linked to negative outcomes amongst youths, such as poor academic performance (Arsenio and Loria, 2014), physical illness (Segerstrom and Miller, 2004), psychological distress (Reisbig et al., 2012), and suicidal ideation (Ang and Huan, 2006b; Park and Chung, 2014). A recent article by Scandurra and colleagues (Scandurra et al., 2024) provided a discussion on the emerging construct of academic psychological distress, and its related environmental antecedents (e.g., others' expectations, academic climate) and consequences (e.g., poorer mental health). A systematic review on academic stress during the final years of secondary school suggested that one in six students experienced excessive distress, with distress increasing as major examinations approached and decreasing as examinations ended (Wuthrich et al., 2020). Higher academic stress was found to be associated with lower academic engagement and motivation, potentially leading to poorer accomplishment and early school dropout, which has implications on successful future employment (Pascoe et al., 2020). Among medical students, numerous studies have been conducted with the goal of assessing the relationship between academic stress and psychological distress. A systematic review (Lyndon et al., 2014) confirmed that academic tests triggered feelings of anxiety, even resulting in higher physiological measures of heart rate and blood pressure.

According to a cross-cultural study (Persike and Seiffge-Krenke, 2012), a comparison of adolescents across Western, Eastern/Asian, and Southern world regions revealed that “problems in the school” and “parents” domains were universally ranked as the highest sources of stress, highlighting the critical influence of these domains on adolescents' wellbeing. The study also examined coping competencies across these three regions and found that two healthier coping styles (negotiating/seeking support; emotional outlet) were used less frequently by those in the Eastern/Asian regions compared to their Western counterparts. The link between academic achievement and stress has been hypothesized to be stronger in Asian cultures; achievement is seen as a mechanism for social mobility and the lack of it appears to lead to feelings of shame that are collectively shared within the family unit (Ang and Huan, 2006b; Yeh and Huang, 1996). The motivation to avoid experiencing such feelings contributes to herculean efforts on the part of school-attending youths to perform academically, and their expectations to do so can originate internally (from the self) or from external influences (from loved ones or authoritative figures) (Ang and Huan, 2006a). Research has shown that parental support and pressure have a stronger influence on academic motivation and anxiety than teacher support and pressure (Song et al., 2015). A study from Korea (Kim and Lee, 2013) found that when adolescents perceived parental love and support to be conditional upon academic performance, even in a close parent-adolescent relationship, they experienced more intense academic stress, which persisted even in subsequent years. Meanwhile, higher self-imposed expectations usually result from higher levels of perfectionism (Rice et al., 2006) or upward comparison among peers (Kwak et al., 2022). When faced with academic stress, increased parental support is associated with healthier coping habits (e.g., strategizing and comfort-seeking), while negative parental interactions predicted increased disengagement coping (e.g., concealment and escape) (Zimmer-Gembeck et al., 2023).

The Academic Expectations Stress Inventory (AESI) (Ang and Huan, 2006a) was developed to measure levels of stress resulting from perceived expectations from parents and teachers, as well as expectations from the self, among middle and high school students. The instrument has been validated in Singapore (Ang and Huan, 2006a), the United States (Ang et al., 2007), Canada (Ang et al., 2009), Iran (Habibi Asgarabad et al., 2021), China, and South Korea (Zhang et al., 2016). Greater academic expectations stress (AES) measured using the AESI has been found to be correlated with higher levels of depression, anxiety, fear of negative evaluation, and emotional exhaustion and cynicism (Ang and Huan, 2006a; Zhang et al., 2016). The AESI has also been a useful tool in assessing the relationship between academic expectation stress and various other constructs, such as adaptive coping (Kao, 2024), academic hardiness (Abdollahi et al., 2020), and suicidal ideation (Obinna et al., 2021).

Given that adolescence and young adulthood is a tumultuous period characterized by complex biopsychosocial transitions and milestones, youths are at an increased propensity to develop mental health issues as compared to other age groups. According to Kessler et al. (2005), most mental health disorders begin between the ages of 14 and 24 years, highlighting the importance of prevention or early intervention programs for youths at this critical developmental milestone. Adding academic stressors to the myriad of existing risk factors may exacerbate the challenges faced by young people who may not have developed effective coping strategies to manage such stress, as seen by the rising numbers of suicidality in recent years. Suicide is one of the leading causes of death among youths in many Asian countries like South Korea, Japan (Kim et al., 2011), or China (Jiang et al., 2018), and Singapore is no exception, with suicide accounting for 38.7% of deaths amongst youths aged 10 to 29 years (Samaritans of Singapore (SOS), 2023). Furthermore, global prevalence of suicidal ideation is estimated at 14.3–22.6% and 4.6–15.8% for suicide attempts in youth under age 22 (Van Meter et al., 2023). It is thus unsurprising that a majority of suicide prevention efforts focus on addressing academic stress in youths, with programs ranging from awareness to skills training (Katz et al., 2013).

The relationship between academic stress and suicidality has been explored in previous literature. A recent systematic review reported positive associations between academic pressure and depressive and anxiety symptoms, as well as suicidal ideation (Ang and Huan, 2006b; Steare et al., 2023). There was also some evidence for increased service use and suicide attempts and rates during the school term as compared to during school holidays and term breaks (Blackburn et al., 2021; Matsubayashi et al., 2016; Steare et al., 2023). By examining the sources of academic stress separately, intervention efforts can be more productively channeled into appropriate avenues. For self-imposed stress, concepts such as self-compassion and cognitive restructuring may be more pertinent (Allen and Leary, 2010); for other-imposed expectations, it may be more applicable to promote effective coping skills (Kao, 2024) or target parent/teacher behavior. Thus, the objectives of the present study are to: (1) describe and compare AESI total and subscale scores among youths aged 15–35 years currently studying in Singapore; (2) investigate relevant sociodemographic correlates; and (3) examine the association between AESI scores and symptoms of depression and anxiety, with a brief exploration on suicidality. It is hoped that results from this study would inform future interventions targeted at managing academic expectations and preventing suicide among youth, as well as identifying potential risk or protective factors of parents or the youth themselves. Possible mechanisms and implications from the findings will also be discussed.

## 2 Materials and methods

Data analyzed for this study was collected as part of a larger study - the National Youth Mental Health Study (Subramaniam et al., 2025), a cross-sectional nationwide survey of youths aged 15–35 years old. This study was approved by the National Healthcare Group's Domain Specific Review Board (DSRB Ref. No.: 2021-00562). Conducted in 2023, the survey aimed to establish the prevalence and correlates of various mental health issues among young people in Singapore. Participants had to meet the following inclusion criteria: (a) aged between 15–35 years, (b) Singapore citizens or permanent residents, (c) literate in English, Chinese, Bahasa Melayu, or Tamil. Participants were recruited through door knocks and street intercepts, following a quota plan developed to ensure an adequate sample size by gender, age, ethnicity, and geographical district. Written informed consent was obtained from all participants (as well as legal guardians of participants aged < 21 years) prior to their enrolment in the study. All procedures were conducted in accordance with the Declaration of Helsinki.

The survey contained a battery of self-administered instruments, including a sociodemographic section, the Depression Anxiety Stress Scales 21 (DASS-21) (Lovibond and Lovibond, 1995), and the AESI. Sociodemographic information collected include age, gender, ethnicity, monthly household income, current educational level, parents' highest educational level attained, and number of siblings. The DASS-21 includes 21 items measuring depression (e.g., “*I felt down-hearted and blue*”), anxiety (e.g., “*I felt I was close to panic*”), and stress (e.g., “*I found it difficult to relax*”) levels over the past week, with higher scores indicating greater severity of symptoms. Cutoff scores of 21 and above for depression and 20 and above for anxiety were categorized as having severe symptoms and were used for analysis for this study (Lovibond and Lovibond, 1995). The performance of the DASS-21 in the current study had been reported previously in an earlier publication (Abdin et al., 2025). The AESI was administered only to participants who indicated “Yes” on whether they were currently studying. The AESI is made up of nine statements, each rated on a five-point scale (from “*never true*” to “*almost always true*”), and consists of two subscales–perceived expectations of parents/teachers (e.g., “*I feel stressed when I know my parents are disappointed in my exam grades*”, “*I feel I have disappointed my teacher when I do badly in school*”), and expectations of self (e.g., “*I feel stressed when I do not live up to my own standards*”). Cronbach's alphas for the AESI scale and self and parents/teachers subscales were 0.945, 0.923, and 0.934, respectively, indicating excellent internal reliability. Scores were obtained by summing the relevant items for each subscale and a total score was obtained by summing all items in the scale. Scores for the AESI total and self and parents/teachers subscales ranged from 9 to 45, 5 to 25, and 4 to 20, respectively, with higher scores indicating greater stress from academic expectations (Ang and Huan, 2006a). Nearing the tail-end of the study, a brief questionnaire on suicidality was added into the survey, in line with increasing concerns on its rising prevalence in the nation. Participants indicated (“*yes*” or “*no*”) whether they have had the following experiences in the previous 12 months: (a) serious thoughts of committing suicide; (b) making a plan for committing suicide; and (c) making a suicide attempt. Suicidal ideation or attempt was dichotomized and scored as “yes” for participants who indicated “yes” in any of the three questions.

Descriptive statistics were generated to describe the performance of the AESI and its subscales in the subset of participants who reported to be “currently studying”. Independent *t*-tests, one-way ANOVAs, and multiple linear regressions were conducted to identify sociodemographic correlates of AESI scores. Bonferroni correction was applied to adjust for multiple comparisons. Logistic regressions were also used to examine if AESI scores were associated with severe depression and anxiety as scored on the DASS-21. Additionally, among those who were administered the questionnaire on suicidality, independent *t*-tests and chi-square tests were conducted to compare sociodemographic data and AESI scores between those who endorsed having serious thoughts of committing suicide and those who did not. A logistic regression was conducted to preliminarily explore the association between AESI and suicidal ideation. Statistical significance was established at *p* < 0.05. All statistical analyses were conducted using Statistical Analysis Software version 9.4 (SAS Institute, Cary, NC).

## 3 Results

**Figure 1:**
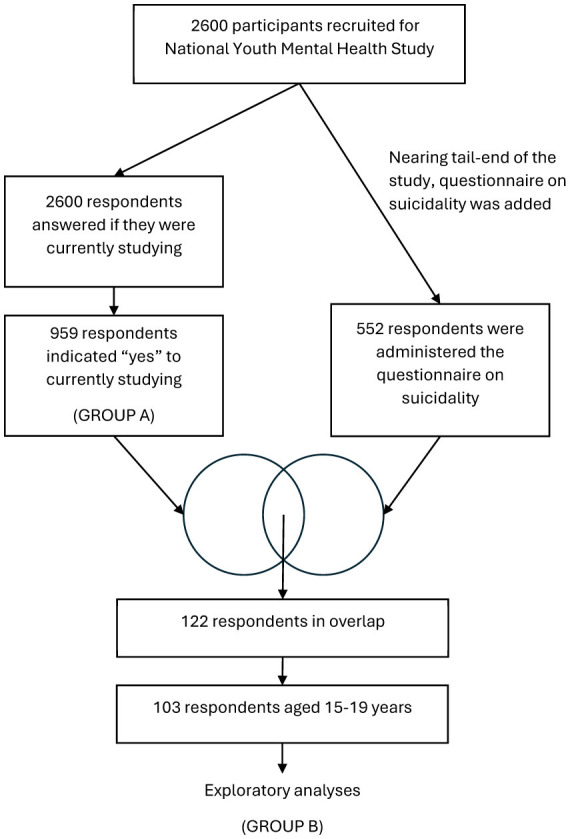
Flow diagram of study participants included in statistical analyses.

Overall, 1948 (76.3%) participants were recruited by household sampling and 652 (23.7%) hard-to-reach youths were recruited via street intercept. Of the total 2600 participants who were recruited for the original study, 959 respondents indicated that they were “currently studying” (Figure 1: Group A), with 227 (23.7%) undergoing secondary school, 398 (41.5%) pursuing post-secondary education, and 334 (34.8%) attending university or higher education. Sociodemographic characteristics of the sample are reported in Table 1. The mean (SD) scores of the overall AESI and the “expectations of parents/teachers” and “expectations of self” subscales were 27.7 (10.0), 14.6 (6.1), and 13.1 (4.8), respectively. Results of the multiple linear regressions of the AESI and its subscales are included in Table 2.

**Table 1:** Sociodemographic correlates of the AESI and its subscales.

Sociodemographic variables	*N* (%)	AESI total		Expectations of parents/teachers subscale		Expectations of self subscale	
		Mean (SD)	*P* value/Adjusted *P* value	Mean (SD)	*P* value/Adjusted *P* value	Mean (SD)	*P* value/Adjusted *P* value
Age group							
15–19	553 (57.7)	28.4 (10.3)	0.001^**^/0.001^**^	15.3 (6.2)	< 0.001^**^/0.001^**^	13.1 (4.8)	0.106/1.000
20–24	301 (31.4)	27.4 (9.5)		14 (5.6)		13.4 (4.7)	
25–29	80 (8.3)	25.4 (9.7)		12.8 (5.9)		12.7 (4.6)	
30–35	25 (2.6)	21.8 (8.4)		10.7 (4.8)		11.1 (4)	
Gender							
Female	431 (44.9)	29.8 (9.8)	< 0.001^*^/0.001^**^	15.6 (6.1)	< 0.001^**^/0.001^**^	14.2 (4.6)	< 0.001^**^/0.001^**^
Male	528 (55.1)	26 (9.8)		13.7 (5.9)		12.3 (4.7)	
Ethnicity							
Chinese	476 (49.6)	27.6 (9.7)	0.023^*^/0.483	14.3 (5.9)	0.014^*^/0.294	13.2 (4.6)	0.064/1.000
Malay	212 (22.1)	27.8 (10)		14.8 (6)		13 (4.9)	
Indian	226 (23.6)	27.1 (10.8)		14.3 (6.4)		12.8 (5)	
Others	45 (4.7)	32.1 (8.7)		17.3 (5.6)		14.8 (3.9)	
Monthly household income							
Below S$5,000	403 (42.0)	27.2 (10.1)	0.207/1.000	14.4 (6)	0.435/1.000	12.8 (4.9)	0.081/1.000
S$5,000 to S$9,999	289 (30.1)	27.4 (10.3)		14.3 (6.2)		13.1 (4.8)	
S$10,000 to S$19,999	189 (19.7)	28.9 (9.4)		15.1 (5.8)		13.8 (4.5)	
S$20,000 and above	78 (8.1)	28.5 (9.9)		15.1 (6.3)		13.4 (4.4)	
Mother's education							
Primary and below	138 (14.5)	26 (9.8)	0.099/1.000	13.4 (6)	0.034^*^/0.714	12.6 (4.7)	0.205/1.000
Secondary	257 (27.0)	28 (10.3)		14.8 (6.1)		13.2 (4.9)	
Post secondary	266 (28.0)	27.3 (10.3)		14.4 (6.1)		12.9 (4.9)	
University & above	290 (30.5)	28.7 (9.4)		15.1 (5.9)		13.6 (4.4)	
Father's education							
Primary and below	104 (11.0)	28.1 (9.4)	0.056/1.000	14.6 (5.7)	0.164/1.000	13.5 (4.7)	0.042^*^/0.882
Secondary	221 (23.4)	27.5 (10.1)		14.6 (6)		12.9 (4.7)	
Post secondary	329 (34.8)	26.9 (10.4)		14.1 (6.2)		12.7 (5)	
University & above	292 (30.9)	29 (9.5)		15.2 (5.9)		13.7 (4.5)	
Number of siblings							
0	125 (13.0)	28.2 (10.4)	0.099/1.000	14.7 (6.4)	0.404/1.000	13.5 (4.9)	0.018^*^/0.378
1	385 (40.1)	28.4 (10)		14.9 (6)		13.6 (4.7)	
2 or more	449 (46.8)	27 (9.9)		14.3 (6)		12.7 (4.7)	

^*^p < 0.05; ^**^p < 0.01.

**Table 2:** Associations between sociodemographic variables and AESI.

Sociodemographic variables	AESI total				Expectations of parents/teachers subscale				Expectations of self subscale			
	B	95% CI	*P* value	VIF	B	95% CI	*P* value	VIF	B	95% CI	*P* value	VIF
Age group												
15–19	8.03	4.03–12.02	< 0.001^*^	1.1	5.31	2.88–7.74	< 0.001^**^	1.1	2.72	0.81–4.62	0.005^**^	1.1
20–24	7.29	3.23–11.34	0.004^*^	1.1	4.23	1.77–6.69	0.013^*^	1.1	3.06	1.13–4.99	0.002^**^	1.1
25–29	5.45	1.04–9.86	0.015^*^	1.1	3.03	0.35–5.71	0.002^**^	1.1	2.42	0.32–4.52	0.024^*^	1.1
30–35	Ref.				Ref.				Ref.			
Gender												
Female	3.77	2.52–5.03	< 0.001^**^	1.0	1.77	1.01–2.54	< 0.001^**^	1.0	2.00	1.40–2.59	< 0.001^**^	1.0
Male	Ref.				Ref.				Ref.			
Ethnicity												
Chinese	Ref.				Ref.				Ref.			
Malay	1.77	0.02–3.51	0.047^*^	1.4	1.21	0.15–2.27	0.025^*^	1.4	0.56	−0.27–1.39	0.189	1.4
Indian	−0.19	−1.77–1.40	0.819	1.2	0.11	−0.85–1.08	0.817	1.2	−0.30	−1.05–0.46	0.437	1.2
Others	4.18	1.14–7.21	0.007^**^	1.1	2.70	0.86–4.54	0.004^**^	1.1	1.47	0.03–2.92	0.045^*^	1.1
Monthly household income												
Below S$5,000	Ref.				Ref.				Ref.			
S$5,000 to S$9,999	0.55	−0.99–2.10	0.481	1.3	0.19	−0.75–1.12	0.697	1.3	0.37	−0.37–1.10	0.326	1.3
S$10,000 to S$19,999	2.12	0.24–4.00	0.028^*^	1.5	0.98	−0.16–2.13	0.091	1.5	1.13	0.24–2.03	0.013^*^	1.5
S$20,000 and above	1.82	−0.7–4.35	0.157	1.3	1.16	−0.37–2.69	0.138	1.3	0.66	−0.54–1.87	0.278	1.3
Mother's education												
Primary and below	−0.91	−3.48–1.66	0.486	2.1	−0.76	−2.32–0.8	0.337	2.1	−0.15	−1.37–1.07	0.811	2.1
Secondary	0.60	−1.44–2.64	0.564	2.2	0.30	−0.93–1.54	0.630	2.2	0.30	−0.67–1.27	0.550	2.2
Post secondary	−0.37	−2.19–1.45	0.691	1.8	−0.23	−1.33–0.88	0.687	1.8	−0.14	−1.01–0.73	0.748	1.8
University and above	Ref.				Ref.				Ref.			
Father's education												
Primary and below	1.31	−1.44–4.05	0.35	1.9	0.81	−0.85–2.48	0.339	1.9	0.49	−0.81–1.80	0.458	1.9
Secondary	−0.77	−2.90–1.37	0.482	2.2	−0.24	−1.54–1.05	0.713	2.2	−0.52	−1.54–0.49	0.313	2.2
Post secondary	−1.80	−3.55–−0.06	0.043^*^	1.8	−0.93	−1.99–0.13	0.085	1.8	−0.87	−1.70–−0.04	0.040^*^	1.8
University and above	Ref.				Ref.				Ref.			
Number of siblings												
0	Ref.				Ref.				Ref.			
1	−0.98	−2.99–1.03	0.338	2.6	−0.56	−1.78–0.66	0.370	2.6	−0.42	−1.38–0.53	0.385	2.6
2 or more	−2.51	−4.52–−0.51	0.014^*^	2.6	−1.21	−2.43–0.01	0.051	2.6	−1.30	−2.26–0.35	0.008^**^	2.6
*R*^2^ value	0.09	0.08	0.08

^*^p < 0.05; ^**^p < 0.01.

On the AESI total scale, age group, gender, ethnicity, monthly household income, paternal education, and number of siblings remained significant correlates in the regression, after accounting for other sociodemographic variables (Table 2). For the self subscale, these same six variables were significant, while only age group, gender, and ethnicity remained significant on the parents/teachers subscale. Specifically, higher scores on the parents/teachers subscale were associated with the 15–19 (vs. the rest) age group, female gender, and Malay or Others (vs. Chinese) ethnicities. Additionally, other than belonging to the 15–19 age group (vs. 30–35), female gender, Others ethnicity (vs. Chinese), higher scores on the self subscale were also associated with monthly household income of S$10,000–19,999 (vs. below S$5,000), paternal education of university and above (vs. post-secondary), and being an only child (vs. having two or more siblings). To account for fundamental differences across developmental stages, regressions were conducted separately for the different age groups (15–19, 20–24, 25–29, and 30–35 years). These results are included as Supplementary material. Gender remained significantly associated with higher AESI scores among age groups 15–19, 20–24, and 30–35 years, but not in age group 25–29 years. Household income was significant in age groups 15–19 and 25–29 years, and number of siblings in age group 20–24 years only. Logistic regression analyses indicated that AESI scores were significantly associated with severe depression and anxiety (DASS-21 cutoff scores), even after adjusting for sociodemographic factors. Coefficients and significance values are presented in Table 3. Similarly, separate results for each age group has been included as Supplementary material. While there were no substantial differences across the age groups in terms of the association between AESI and severe levels of depression and anxiety, two associations with severe depression became non-significant after age stratification—the self subscale in age group 20–24 years, and the parents/teachers subscale in age group 25–29 years.

**Table 3:** Odds ratios from the regressions between DASS-21 and AESI.

Academic expectations stress inventory	DASS-21 Depression subscale				DASS-21 Anxiety subscale			
	OR	95% CI	*P* value	*R*^2^ value	OR	95% CI	*P* value	*R*^2^ value
AESI total	1.09	1.07–1.12	< 0.001^**^	0.12	1.09	1.07–1.11	< 0.001^**^	0.19
Self subscale	1.18	1.13–1.24	< 0.001^**^	0.10	1.17	1.13–1.22	< 0.001^**^	0.17
Parents/teachers subscale	1.14	1.10–1.18	< 0.001^**^	0.11	1.13	1.10–1.16	< 0.001^**^	0.17

^**^p < 0.01.

Analysis ran was corrected for sociodemographic factors namely, age, gender, ethnicity, household income, parents' education and number of siblings.

The suicidality questionnaire, which was added to the National Youth Mental Health Study at a later stage, was administered to 552 participants. Of these, 63 (11.4%) endorsed experiencing at least one suicidal ideation or attempt in the past 12 months. Focusing on the subset of 122 participants who were currently studying, the majority (103 participants) were aged 15–19 years old, and as such only data from respondents who belonged to this age group were included for subsequent exploratory analyses (Figure 1: Group B). 25 (24.3%) of these participants answered “yes” to experiencing at least one suicidal ideation or attempt in the past 12 months. There was no significant difference in gender in terms of whether these participants endorsed at least one suicidal ideation or attempt, χ^2^(1, *N* = 103) = 0.793, *p* = 0.373. Meanwhile, independent *t*-tests showed that those who did (*M* = 33.4, *SD* = 9.2) tended to score higher on the AESI total, *t*(101) = 2.77, *p* = 0.007, than those who did not (*M* = 27.3, *SD* = 9.7). They (*M* = 18.7, *SD* = 6.1) also scored higher on the expectations of parents/teachers subscale *t*(101) = 3.08, *p* = 0.003, than those who did not endorse any suicidal ideation or attempts (*M* = 14.4, *SD* = 6.1), but not on the expectations of self subscale, *t*(101) = 1.67, *p* = 0.099. Due to the small sample size, only gender was controlled for in the logistic regression, which also revealed that the AESI total score remained significantly associated with having at least one suicidal ideation or attempt in the past 12 months (OR = 1.095, 95% CI = 1.03–1.16, *p* = 0.003).

## 4 Discussion

The goal of the present study was to provide some insights into AES experienced by youths aged 15–35 years, by analyzing data collected from a cross-sectional nationwide study in Singapore. The significant associations reported between the DASS-21 depression and anxiety and AESI scores have further highlighted the negative implications of AES amongst those currently studying. Sociodemographic factors associated with stress rated on the AESI varied slightly between the two subscales, with age, gender, and ethnicity found to be associated with perceived expectations from parents/teachers, and the addition of household income, paternal education, and presence of siblings associated with expectations from self.

Female respondents in the current study reported significantly higher perceived AES, regardless of whether it came from parents/teachers or self. This is congruent with previous research, where gender differences in the experience of academic stress have been long established, including perceived stress and coping (Graves et al., 2021; Liu and Lu, 2012) and academic achievement (Voyer and Voyer Susan, 2014). A possible reason for this could be that female students are more likely to internalize problems and expectations as compared to their male peers (Gutman and Codiroli McMaster, 2020). Female students have also been found to be more prone to higher levels of resulting mental distress (Chyu and Chen, 2022; Dalen, 2014), even if they outperform their male classmates (Pomerantz et al., 2002). Additionally, in the cross-cultural study by (Persike and Seiffge-Krenke 2012), females from all three world regions were more likely to report higher levels of all coping styles, including withdrawal/denial, which was concerning as withdrawal/denial has been shown to lead to increased psychopathology (Seiffge-Krenke and Stemmler, 2002). Taken together, the evidence suggests that the developmental significance of perceived academic competence differs between male and female students, with poor academic achievement more likely to result in depressive symptoms in females (Yong et al., 2022).

The finding in the present study that belonging to the middle-income bracket is a significant predictor of higher AES by self is consistent with existing literature. A study by Ponnet (2014) demonstrated that there was a direct effect of paternal financial stress on positive parenting in middle-income families, but not in low- or high-income families, which lends support to the hypothesis that families across different income brackets experience different pathways of expressing and experiencing stress. For youths in lower income families, the risk of being exposed to traumatic or stressful life situations is higher, which may result in the prioritization of more immediate needs outside of school performance (Reiss et al., 2019). Parents in low-income families may also be more focused on overcoming financial challenges rather than exerting academic pressure on their children. Conversely, children in higher income families are described to have increased access to educational resources and higher self-efficacy, and hence lower test anxiety (Xu et al., 2021). Hence, for students from middle-income families, the lack of financial security (that high-income families enjoy) or financial aid (that low-income families receive), compounded with the stronger perceived need for upward social mobility through academic achievement, would exert significant pressure on them to succeed, especially in a meritocratic society such as Singapore's. Belonging to a middle-income family as opposed to low- or high-income families would also bring one's position on the socioeconomic ladder into sharper focus, and comparison of lifestyles among peers may result in increased anxiety to maintain their socioeconomic status (Eriksen, 2021).

Being an only child is often linked to increased parental involvement and scrutiny. An only child would not need to compete with siblings for parental attention nor would the parents need to provide social support for multiple siblings (Khadaroo and MacCallum, 2021). Without siblings to dilute parental expectations or provide emotional support, only children are more likely to develop perfectionistic tendencies through increased sensitivity and internalization of these expectations (Xu et al., 2021), which explains the finding of only children in the current study having higher academic expectations on themselves. On the other hand, some results from the present study diverged from past literature. A study in India found that children of fathers with lower education were more likely to perceive parental pressure for better academic performance (Deb et al., 2015), while a study in Germany reported that students preparing for vocational careers were more susceptible to paternal pressure and students preparing for university education were more susceptible to maternal pressure (Kulakow et al., 2021). In contrast, while scores on the AESI parents/teachers subscale in the present study were originally significantly different across the levels of maternal education (highest among those with maternal education of university and above), this finding was not significant in the regression model after accounting for the other sociodemographic factors. Meanwhile, paternal education of university and above predicted higher scores on the AESI self subscale. It is likely that this was due to age or cultural differences in the study population, and warrants a more systematic investigation to produce more robust findings.

Within respondents aged 15–19 years, perceiving greater AES from parents/teachers was associated with at least one instance of suicidal ideation or behavior, a finding that was not replicated with expectations from self. This seems to preliminarily suggest that academic expectations from others could be a potential moderator of the relationship between experienced stress and suicidality. Indeed, a qualitative study on middle-income youth in Norway narrated how explicit parental demands were tied to mental health symptoms, such as hopelessness and struggling with negative emotions (Eriksen, 2021). Suicidal ideation has also been associated with family communication patterns and expectations of perfection (Miller and Day, 2002). However, another study in India found the opposite—that academic expectations from self were related to suicidal ideation, but not expectations from others (Ortiz et al., 2023). More studies with consistent findings are needed before conclusive statements on the relationship between expectations from others and suicidality can be drawn.

The main limitation of the current study was that suicidality data was not collected across all participants, as the questionnaire was only included after the survey was underway. Therefore, the analysis was limited to only respondents within the age range of 15–19 years, which may not be representative of the entire youth population. Furthermore, the measurement used was a brief questionnaire with only dichotomous yes/no response options and relied heavily on retrospective participant recall. The suicidality questionnaire measured suicidal ideation and behavior in the past 12 months, while the DASS-21 measured depression and anxiety symptoms for the past week only. This limits the precision and generalizability of the suicidality findings, which should be interpreted with considerable caution. However, given the heightened concern about youth suicidality during the study period, the study team considered it valuable to include these exploratory findings to supplement preliminary insights for future research and provide an estimation of the prevalence of suicidal behavior in Singaporean youths. Secondly, given that this was a national survey with broad recruitment objectives, the sampling strategy was not intentionally designed for equal representation across age groups of those currently studying. As a result, the age groups of 25–29 and 30–35 years had substantially smaller sample sizes as compared to age groups 15–19 and 20–24 years, resulting in reduced statistical power for detecting meaningful associations in the age-stratified analyses. Nevertheless, the variation observed across the separate analyses potentially suggests that sociodemographic factors may function differently as risk or protective factors across different life stages or developmental contexts. Future research would benefit from purposive sampling strategies to ensure sufficient statistical power to support rigorous analytical approaches. Another limitation was that information on teachers and the school and/or classroom climate was not collected. Studies have indicated that factors such as student-teacher relationships, availability of support for teachers, or even methods of learning could influence students' resilience levels or their perceptions of teachers' expectations to be motivational or stressful (Jia and Cheng, 2024; Podiya et al., 2025). More empirical studies are required to investigate how this stress from expectations of others can be effectively managed on a personal or system level, but already there are some promising findings on cognitive-behavioral therapies delivered by teachers for students (Jagiello et al., 2024) and mindfulness-based approaches for teachers, among others (von der Embse et al., 2019). Lastly, while the results may contribute to and validate existing international literature on AES, the generalizability of these findings to other cultural contexts remains to be established, as the influence of Singapore's cultural and educational landscape was not accounted for in the present study.

## 5 Conclusions

This study's findings highlighted sociodemographic factors that could be potential risk or protective factors for AES among youths, while preliminarily exploring the relationship between sources of this stress with suicidality. In sum, the data seems to suggest that being female, being an only child, and belonging to the middle-income group are associated with increased risk for greater academic stress, particularly due to expectations from the self. The broad coverage of the survey provided a systematic evaluation of AES experienced by young people aged 15–35 years old in Singapore who are currently studying. Close and continued monitoring of these stress levels nationwide could provide crucial information on the effectiveness of existing mental health programs and aid in guiding early intervention strategies.

## Data Availability

The data used in this study will be made available upon reasonable request to the corresponding author, as per the institutional data access policies. Requests to access the datasets should be directed to Chua Yi Chian, yi.chian.chua@nhghealth.com.sg.
