# Supplementary material for: Academic expectations stress, its associations with symptoms of depression and anxiety, and preliminary exploration of suicidality: results from a National Youth Mental Health Survey in Singapore
Source: Front Psychol. 2026 May 28;17:1785980. doi: 10.3389/fpsyg.2026.1785980 (PMC13253745; doi:10.3389/fpsyg.2026.1785980)
Supplement: Supplementary file 1 [file Table_1.docx]

Associations between sociodemographic variables and AESI for age group 15-19 years (n=553).

|  | AESI total | | | | Expectations of  parents/teachers subscale | | | | Expectations of  self subscale | | | |
| --- | --- | --- | --- | --- | --- | --- | --- | --- | --- | --- | --- | --- |
|  | B | 95% CI | P value | VIF | B | 95% CI | P value | VIF | B | 95% CI | P value | VIF |
| Gender   - Female - Male | 4.89  Ref. | 3.21-6.57 | <0.001^**^ | 1.0 | 2.44  Ref. | 1.40-3.47 | <0.001^**^ | 1.0 | 2.45  Ref. | 1.67-3.23 | <0.001^**^ | 1.0 |
| Ethnicity   - Chinese - Malay - Indian - Others | Ref.  1.25  1.09  1.99 | -1.09-3.58  -2.77-1.52  -0.86-6.96 | 0.295  0.569  0.126 | 1.5  1.3  1.1 | Ref.  0.95  -0.25  1.92 | -0.49-2.38  -1.57-1.07  -0.48-4.32 | 0.197  0.714  0.117 | 1.5  1.3  1.1 | Ref.  0.30  -0.38  1.13 | -0.79-1.39  -1.38-0.62  -0.69-2.95 | 0.587  0.460  0.224 | 1.5  1.3  1.1 |
| Monthly household income   - Below S$5,000 - S$5,000 to S$9,999 - S$10,000 to S$19,999 - S$20,000 and above | Ref.  0.45  1.88  4.58 | -1.65-2.55  -0.69-4.44  0.87-8.28 | 0.672  0.151  0.016^*^ | 1.3  1.4  1.2 | Ref.  0.33  0.65  2.90 | -0.96-1.62  -0.92-2.23  0.62-5.18 | 0.614  0.417  0.013^*^ | 1.3  1.4  1.2 | Ref.  0.12  1.22  1.67 | -0.86-1.1  0.03-2.42  -0.05-3.4 | 0.809  0.045  0.057 | 1.3  1.4  1.2 |
| Mother's education   - Primary and below - Secondary - Post secondary - University & above | -0.28  0.18  -0.17  Ref. | -4.03-3.47  -2.56-2.91  -2.58-2.25 | 0.884  0.898  0.892 | 1.8  2.2  1.7 | -0.22  0.19  -0.24  Ref. | -2.53-2.09  -1.49-1.88  -1.72-1.25 | 0.851  0.822  0.753 | 1.8  2.2  1.7 | -0.06  -0.01  0.07  Ref. | -1.81-1.69  -1.29-1.26  -1.05-1.20 | 0.948  0.982  0.901 | 1.8  2.2  1.7 |
| Father's education   - Primary and below - Secondary - Post secondary - University & above | 0.78  -1.03  -1.66  Ref. | -3.11-4.68  -3.94-1.88  -3.97-0.66 | 0.693  0.487  0.160 | 1.8  2.2  1.8 | 0.29  -0.28  -0.81  Ref. | -2.11-2.69  -2.07-1.52  -2.23-0.62 | 0.813  0.763  0.267 | 1.8  2.2  1.8 | 0.49  -0.75  -0.85  Ref. | -1.32-2.31  -2.11-0.60  -1.93-0.23 | 0.593  0.275  0.122 | 1.8  2.2  1.8 |
| Number of siblings   - 0 - 1 - 2 or more | Ref.  -0.64  -2.65 | -3.48-2.20  -5.49-0.19 | 0.658  0.068 | 2.8  2.9 | Ref.  -0.57  -1.38 | -2.31-1.18  -3.13-0.36 | 0.522  0.120 | 2.8  2.9 | Ref.  -0.07  -1.26 | -1.39-1.25  -2.59-0.06 | 0.917  0.062 | 2.8  2.9 |
| R^2^ value | 0.10 | | | | 0.07 | | | | 0.12 | | | |
| *p<0.05; **p<0.01 | | | | | | | | | | | | |

Associations between sociodemographic variables and AESI for age group 20-24 years (n=301).

|  | AESI total | | | | Expectations of  parents/teachers subscale | | | | Expectations of  self subscale | | | |
| --- | --- | --- | --- | --- | --- | --- | --- | --- | --- | --- | --- | --- |
|  | B | 95% CI | P value | VIF | B | 95% CI | P value | VIF | B | 95% CI | P value | VIF |
| Gender   - Female - Male | 2.85  Ref. | 0.61-5.10 | 0.013^*^ | 1.0 | 1.35  Ref. | 0.01-2.68 | 0.049* | 1.0 | 1.51  Ref. | 0.41-2.61 | 0.007*  Ref. | 1.0 |
| Ethnicity   - Chinese - Malay - Indian - Others | Ref.  1.65  1.43  2.84 | -1.48-5.02  -1.94-3.67  2.55-13.73 | 0.545  0.286  0.005^**^ | 1.2  1.1  1.1 | Ref.  0.87  0.91  4.49 | -1.07-2.81  -0.76-2.59  1.16-7.83 | 0.377  0.284  0.008** | 1.2  1.1  1.1 | Ref.  0.90  -0.05  3.64 | -0.70-2.49  -1.43-1.33  0.90-6.38 | Ref.  0.269  0.944  0.009 | 1.2  1.1  1.1 |
| Monthly household income   - Below S$5,000 - S$5,000 to S$9,999 - S$10,000 to S$19,999 - S$20,000 and above | Ref.  0.14  0.90  -0.12 | -2.63-2.91  -2.54-4.35  -4.20-3.96 | 0.921  0.606  0.953 | 1.6  1.7  1.4 | Ref.  -0.12  0.36  -0.40 | -1.77-1.54  -1.70-2.41  -2.83-2.04 | 0.888  0.734  0.748 | 1.6  1.7  1.4 | Ref.  0.26  0.55  0.28 | -1.10-1.62  -1.14-2.24  -1.73-2.28 | Ref.  0.708  0.524  0.786 | 1.6  1.7  1.4 |
| Mother's education   - Primary and below - Secondary - Post secondary - University & above | 1.39  1.69  -0.14  Ref. | -2.86-5.65  -1.76-5.15  -3.27-3.00 | 0.519  0.336  0.931 | 2.0  1.9  1.8 | 0.52  0.62  0.20  Ref. | -2.01-3.06  -1.44-2.68  -1.67-2.07 | 0.685  0.555  0.834 | 2.0  1.9  1.8 | 0.87  1.08  -0.34  Ref. | -1.22-2.96  -0.62-2.77  -1.87-1.20 | 0.412  0.213  0.667  Ref. | 2.0  1.9  1.8 |
| Father's education   - Primary and below - Secondary - Post secondary - University & above | -0.65  -0.81  -2.48  Ref. | -5.43-4.14  -4.35-2.74  -5.53-0.56 | 0.790  0.655  0.109 | 1.8  1.9  1.8 | 0.27  -0.65  -1.51  Ref. | -2.59-3.12  -2.76-1.47  -3.32-0.31 | 0.854  0.547  0.103 | 1.8  1.9  1.8 | -0.92  -0.16  -0.98  Ref. | -3.26-1.43  -1.90-1.58  -2.47-0.51 | 0.444  0.857  0.198  Ref. | 1.8  1.9  1.8 |
| Number of siblings   - 0 - 1 - 2 or more | Ref.  -3.21  -3.50 | -6.7-0.28  -6.97--0.04 | 0.071  0.047^*^ | 2.6  2.6 | Ref.  -1.49  -1.43 | -3.57-0.59  -3.49-0.64 | 0.160  0.175 | 2.6  2.6 | Ref.  -1.72  -2.08 | -3.43--0.01  -3.78--0.38 | Ref.  0.049*  0.017* | 2.6  2.6 |
| R^2^ value | 0.08 | | | | 0.06 | | | | 0.09 | | | |
| *p<0.05; **p<0.01 | | | | | | | | | | | | |

Associations between sociodemographic variables and AESI for age group 25-29 years (n=80).

|  | AESI total | | | | Expectations of  parents/teachers subscale | | | | Expectations of  self subscale | | | |
| --- | --- | --- | --- | --- | --- | --- | --- | --- | --- | --- | --- | --- |
|  | B | 95% CI | P value | VIF | B | 95% CI | P value | VIF | B | 95% CI | P value | VIF |
| Gender   - Female - Male | 1.77  Ref. | -2.79-6.34 | 0.441 | 1.1 | 0.82  Ref. | -1.98-3.61 | 0.561 | 1.1 | 0.96  Ref. | -1.16-3.07 | 0.371 | 1.1 |
| Ethnicity   - Chinese - Malay - Indian - Others | Ref.  2.62  -1.34  -2.10 | -2.95-8.20  -7.35-4.68  -18.40-14.20 | 0.658  0.350  0.798 | 1.4  1.5  1.5 | Ref.  1.85  0.02  2.21 | -1.56-5.26  -3.66-3.70  -7.77-12.18 | 0.283  0.990  0.660 | 1.4  1.5  1.5 | Ref.  0.78  -1.36  -4.30 | -1.81-3.36  -4.15-1.43  -11.87-3.26 | 0.551  0.334  0.260 | 1.4  1.5  1.5 |
| Monthly household income   - Below S$5,000 - S$5,000 to S$9,999 - S$10,000 to S$19,999 - S$20,000 and above | Ref.  0.19  4.44  -5.69 | -5.27-5.66  -2.09-10.96  -13.73-2.35 | 0.944  0.179  0.162 | 1.4  1.7  1.4 | Ref.  -0.02  4.24  -1.81 | -3.37-3.32  0.25-8.24  -6.73-3.12 | 0.989  0.038^*^  0.466 | 1.4  1.7  1.4 | Ref.  0.21  0.19  -3.89 | -2.32-2.75  -2.83-3.22  -7.62--0.16 | 0.867  0.898  0.041^*^ | 1.4  1.7  1.4 |
| Mother's education   - Primary and below - Secondary - Post secondary - University & above | -7.28  0.37  -1.61  Ref. | -17.76-3.20  -10.00-10.75  -11.64-8.42 | 0.170  0.943  0.749 | 6.0  5.4  4.2 | -5.17  -0.95  -2.11  Ref. | -11.58-1.25  -7.31-5.40  -8.25-4.03 | 0.112  0.765  0.494 | 6.0  5.4  4.2 | -2.11  1.33  0.50  Ref. | -6.98-2.75  -3.49-6.15  -4.15-5.15 | 0.388  0.583  0.830 | 6.0  5.4  4.2 |
| Father's education   - Primary and below - Secondary - Post secondary - University & above | 7.14  3.56  1.23  Ref. | -1.25-15.53  -4.54-11.65  -5.51-7.97 | 0.094  0.383  0.717 | 2.7  2.9  2.4 | 3.69  3.04  1.15  Ref. | -1.44-8.83  -1.91-8.00  -2.97-5.28 | 0.155  0.224  0.579 | 2.7  2.9  2.4 | 3.45  0.51  0.08  Ref. | -0.44-7.34  -3.24-4.27  -3.05-3.20 | 0.082  0.786  0.960 | 2.7  2.9  2.4 |
| Number of siblings   - 0 - 1 - 2 or more | Ref.  3.25  1.62 | -3.22-9.72  -5.15-8.40 | 0.320  0.634 | 2.3  2.7 | Ref.  1.94  0.06 | -2.02-5.90  -4.09-4.21 | 0.332  0.977 | 2.3  2.7 | Ref.  1.31  1.56 | -1.69-4.31  -1.58-4.71 | 0.387  0.324 | 2.3  2.7 |
| R^2^ value | 0.27 | | | | 0.27 | | | | 0.28 | | | |
| *p<0.05 | | | | | | | | | | | | |

Associations between sociodemographic variables and AESI for age group 30-35 years (n=25).

|  | AESI total | | | | Expectations of  parents/teachers subscale | | | | Expectations of  self subscale | | | |
| --- | --- | --- | --- | --- | --- | --- | --- | --- | --- | --- | --- | --- |
|  | B | 95% CI | P value | VIF | B | 95% CI | P value | VIF | B | 95% CI | P value | VIF |
| Gender   - Female - Male | -10.78  Ref. | -21.16--0.39 | 0.043^*^ | 2.3 | -5.17  Ref. | -10.82-0.48 | 0.069 | 2.3 | -5.60  Ref. | -10.97--0.23 | 0.043^*^ | 2.3 |
| Ethnicity   - Chinese - Malay - Indian - Others | Ref.  0.52  3.33  12.37 | -24.51-25.55  -6.34-13.00  -4.27-29.01 | 0.964  0.461  0.129 | 7.2  1.9  1.7 | Ref.  3.10  2.98  8.11 | -10.51-16.72  -2.28-8.24  -0.95-17.16 | 0.623  0.236  0.074 | 7.2  1.9  1.7 | Ref.  -2.58  0.35  4.27 | -15.52-10.36  -4.65-5.35  -4.34-12.87 | 0.666  0.878  0.295 | 7.2  1.9  1.7 |
| Monthly household income   - Below S$5,000 - S$5,000 to S$9,999 - S$10,000 to S$19,999 - S$20,000 and above | Ref.  4.10  0.37  12.94 | -7.19-15.38  -20.80-21.55  -14.85-40.74 | 0.438  0.969  0.324 | 2.6  7.7  4.8 | Ref.  2.10  -1.15  6.62 | -4.04-8.24  -12.66-10.37  -8.50-21.74 | 0.463  0.829  0.353 | 2.6  7.7  4.8 | Ref.  2.00  1.52  6.32 | -3.84-7.83  -9.43-12.47  -8.05-20.70 | 0.464  0.764  0.350 | 2.6  7.7  4.8 |
| Mother's education   - Primary and below - Secondary - Post secondary - University & above | -3.70  0.87  14.75  Ref. | -37.99-30.58  -34.51-36.25  -22.66-52.15 | 0.815  0.957  0.400 | 23.1  23.2  12.6 | -6.77  -4.25  3.01  Ref. | -25.42-11.88  -23.49-15  -17.34-23.35 | 0.437  0.634  0.749 | 23.1  23.2  12.6 | 3.07  5.12  11.74  Ref. | -14.66-20.80  -13.18-23.42  -7.60-31.08 | 0.708  0.547  0.206 | 23.1  23.2  12.6 |
| Father's education   - Primary and below - Secondary - Post secondary^#^ - University & above | 12.13  4.66  -  Ref. | -16.02-40.28  -20.09-29.41  - | 0.360  0.684  - | 15.5  12.5  - | 10.90  6.37  -  Ref. | -4.42-26.21  -7.09-19.84  - | 0.144  0.316  - | 15.5  12.5  - | 1.23  -1.72  -  Ref. | -13.33-15.79  -14.52-11.08  - | 0.854  0.771  - | 15.5  12.5  - |
| Number of siblings   - 0 - 1 - 2 or more | Ref.  9.03  14.63 | -7.99-26.04  -3.85-33.12 | 0.265  0.108 | 5.7  7.0 | Ref.  3.80  6.74 | -5.45-13.06  -3.32-16.80 | 0.381  0.166 | 5.7  7.0 | Ref.  5.22  7.89 | -3.58-14.02  -1.67-17.45 | 0.216  0.096 | 5.7  7.0 |
| R^2^ value | 0.64 | | | | 0.68 | | | | 0.58 | | | |
| *p<0.05; ^#^N=0 | | | | | | | | | | | | |

Odds ratios from the regressions between DASS-21 and AESI for age group 15-19 years (n=553).

|  | DASS-21 Depression subscale | | | | DASS-21 Anxiety subscale | | | |
| --- | --- | --- | --- | --- | --- | --- | --- | --- |
|  | OR | 95% CI | P value | R^2^ value | OR | 95% CI | P value | R^2^ value |
| AESI total  Self subscale  Parents/teachers subscale | 1.09  1.19  1.14 | 1.06-1.12  1.12-1.26  1.09-1.19 | <0.001^**^  <0.001^**^  <0.001^**^ | 0.12  0.11  0.11 | 1.11  1.21  1.16 | 1.08-1.13  1.14-1.27  1.12-1.21 | <0.001^**^  0.001^**^  <0.001^**^ | 0.23  0.21  0.22 |
| ^**^*p*<0.01 | | | | | | | | |

Odds ratios from the regressions between DASS-21 and AESI for age group 20-24 years (n=301).

|  | DASS-21 Depression subscale | | | | DASS-21 Anxiety subscale | | | |
| --- | --- | --- | --- | --- | --- | --- | --- | --- |
|  | OR | 95% CI | P value | R^2^ value | OR | 95% CI | P value | R^2^ value |
| AESI total  Self subscale  Parents/teachers subscale | 1.11  1.21  1.16 | 1.07-1.15  1.11-1.32  1.09-1.23 | <0.001^**^  0.590  <0.001^**^ | 0.16  0.14  0.15 | 1.04  1.10  1.06 | 1.02-1.07  1.04-1.17  1.01-1.11 | 0.002^**^  <0.001^**^  0.026^*^ | 0.13  0.13  0.12 |
| ^*^*p*<0.05; ^**^*p*<0.01 | | | | | | | | |

Odds ratios from the regressions between DASS-21 and AESI for age group 25-29 years (n=80).

|  | DASS-21 Depression subscale | | | | DASS-21 Anxiety subscale | | | |
| --- | --- | --- | --- | --- | --- | --- | --- | --- |
|  | OR | 95% CI | P value | R^2^ value | OR | 95% CI | P value | R^2^ value |
| AESI total  Self subscale  Parents/teachers subscale | 1.07  1.08  1.14 | 0.94-1.23  0.82-1.41  0.94-1.39 | <0.001^**^  <0.001^**^  0.187 | 0.28  0.26  0.29 | 1.19  1.35  1.31 | 1.05-1.36  1.05-1.73  1.06-1.62 | 0.008^**^  0.020^*^  0.013^*^ | 0.42  0.38  0.42 |
| ^*^*p*<0.05; ^**^*p*<0.01 | | | | | | | | |

Odds ratios from the regressions between DASS-21 and AESI for age group 30-35 years (n=25).

|  | DASS-21 Depression subscale | | | | DASS-21 Anxiety subscale | | | |
| --- | --- | --- | --- | --- | --- | --- | --- | --- |
|  | OR | 95% CI | P value | R^2^ value | OR | 95% CI | P value | R^2^ value |
| AESI total  Self subscale  Parents/teachers subscale | 2.18  6.74  3.34 | 1.95-2.42  5.14-8.84  2.54-4.38 | <0.001^**^  <0.001^**^  <0.001^**^ | 0.63  0.63  0.63 | 2.30  17.56  3.69 | 2.04-2.60  10.61-29.07  2.96-4.61 | <0.001^**^  <0.001^**^  <0.001^**^ | 0.67  0.67  0.67 |
| ^*^*p*<0.05; ^**^*p*<0.01 | | | | | | | | |
